# HIV Epidemic in Tanzania: The Possible Role of the Key Populations

**DOI:** 10.1155/2017/7089150

**Published:** 2017-08-17

**Authors:** Bonaventura C. T. Mpondo, Daniel W. Gunda, Semvua B. Kilonzo

**Affiliations:** ^1^Department of Internal Medicine, School of Medicine, College of Health Sciences, The University of Dodoma, Dodoma, Tanzania; ^2^Department of Internal Medicine, School of Medicine, Catholic University of Health and Allied Sciences, Mwanza, Tanzania

## Abstract

HIV remains a public health concern in Tanzania and other Eastern and Southern African countries. Estimates show that there were about 1.4 million people living with HIV in Tanzania in the year 2013. HIV is a generalized epidemic in Tanzania with heterosexual transmission being the main route of transmission. Recently, however, there has been growing concern on the potential role of the key populations in HIV epidemic in the country. Studies done have shown significantly higher HIV prevalence in these populations compared to the general population. These studies have also reported high risky behaviors among members of these populations. This review aims at discussing the possible role of the key populations in the HIV epidemic in Tanzania.

## 1. Background

HIV remains a public health concern in many sub-Saharan African countries including Tanzania. In the year 2013, it was estimated that there were a total of 1.4 million people living with HIV in Tanzania [1]. The national prevalence among people aged 15–49 years in Tanzania was estimated to be 5.1% in 2011, which is a drop of approximately 2% compared to the year 2003 [2]. In Tanzania, HIV is a generalized epidemic affecting both urban and rural populations with approximately over 80% of HIV infections resulting from heterosexual transmission [3].

Key populations (KPs) are defined by World Health Organization (WHO) as both vulnerable populations and populations at higher risk of acquiring HIV infection [4]. They usually have both legal and social issues related to their behaviors which increase their vulnerability to HIV. Members of the KPs include people who inject drugs (PWID), men who have sex with men (MSM), transgender persons, female sex workers (FSW), and prisoners. KPs are important to the dynamics of HIV transmission in a given setting and are essential partners in an effective response to the epidemic [5, 6]. There is evidence of overlapping sexual network between KPs and general population [7]. This indicates that HIV among key populations is not isolated; if not properly addressed it could risk the national responses.

Research worldwide shows that HIV disproportionately affects members of the key populations (KPs) as compared to the general population. When compared to the general population, global data shows that on average PWID are 22 times more likely to be HIV-positive [1], transgender 49 times [8], sex workers 14 times [9], MSM 13 times [10, 11], and prisoners 6–50 times, depending on specific contexts [8]. Despite the small number of studies done in Tanzania, evidence indicates HIV prevalence of 42% among PWID [12], 31.4% among female sex workers [13], and 30.2% among MSM [14]. HIV prevalence among transgender people is not known and official data from prisons is not available.

Despite the high HIV prevalence in these populations, there are also reports of multiple sexual partners and high frequency of partner change with low condom usage in these populations [13, 15–17]. According to the sexually transmissible infections (STIs) transmission model as described by Anderson, people who have high rates of partner change have high chances of transmitting HIV and other STIs [18]. These individuals with high frequency change of sexual partners and other risky behaviors form the “core group” with their sexual partners forming the “adjacent group”; this group can serve as “bridging population” between the core group and the general population. This mini-review aims at highlighting the possible role of KPs in HIV epidemic in Tanzania.

## 2. Estimates on Key Population Size in Tanzania

In April 2014, the Ministry of Health of Tanzania together with the Tanzania Commission for AIDS (TACAIDS) met with stakeholders to estimate the HIV prevalence and population estimates of the key populations in mainland Tanzania. The stakeholders reviewed the existing evidence and identified the limitations and knowledge gaps in the studies. They then used a Delphi method [19] to come to a consensus on the estimated population size and HIV prevalence on the three groups of key populations: female sexual workers, men who have sex with men, and people who use/inject drugs [20].

For the female sex workers (FSWs), the population size was estimated to be 155,450 individuals (range: 128,610–198,050). The HIV prevalence in this group was estimated to be 26 percent (range: 14–37%). For men who have sex with men, the population in urban mainland Tanzania was estimated to be 49,700 individuals (range: 41,000–71,000); the HIV prevalence was estimated to be 25 percent (range: 18–35%). Among the people who use/inject drugs, their number was estimated to be 30,000 (range: 20,000–42,500), with a consensus point prevalence estimate of 36 percent (range: 22–43%). Estimates done in Unguja island in Zanzibar in 2011/2012 using multiple methods concluded that there were 3000 PWID, 3958 FSW, and 2157 MSM [21]; the total population of Unguja was estimated to be 896,721 from the 2012 census.

Tanzania is, however, one of the countries where all these populations are criminalized. There is therefore high possibility of the population size to be underestimated; this concept has been proved in recent studies [22].

## 3. Epidemiology of HIV among KPs

### 3.1. Prevalence of HIV among MSM

MSM remain a hidden population in Tanzania because the practice is still stigmatized and criminalized. There are few reports on the possible contribution of MSM in the HIV epidemic in the country. Few studies have been done to determine the magnitude of HIV among MSM in Tanzania. In 2012, a study done in Dar es Salaam and Tanga, two regions along the coast, found that HIV prevalence was 30.2% in Dar es Salaam and 11% in Tanga [14]. In Zanzibar, an island where the prevalence in the general population is estimated to be 0.6%, a survey done in 2007 found the prevalence among the MSM to be 12.3%, which is about 20-fold higher [23]. A recent published study found that the HIV seroprevalence of this group in Dodoma which is the capital city of the country was 17%, which is approximately five times that of men in the general population [24].

### 3.2. Prevalence of HIV among Female Sex Workers

Tanzanian law criminalizes sex work practice and it is punishable by law. Despite this fact, the practice has been documented in Tanzania, especially in Dar-es-Salaam which is the economic capital of the country [13]. Globally, it is estimated that FSW are 14 times more likely to be living with HIV compared to women in the general population [9]. In Tanzania, however, data on HIV prevalence among sexual workers is scarce. Few studies have been done to estimate the magnitude of HIV in this population. A survey conducted in Dar es Salaam in 2010 reported HIV prevalence among sexual workers to be 31.4% [13] compared to the prevalence of 10.4% in the general population [2]. Another study done in the northern part of the country among hotel and bar workers who in most cases are also sexual workers found HIV prevalence of 26.3% [25]. The findings in these studies are in line with the findings from a review that reported high prevalence of around 36.9% in SSA which was the highest compared to other geographical regions [9]. Studies done elsewhere in Africa have reported a wide range of HIV prevalence among FSW: from 34% in Cameroon to 75% in Kenya [26].

### 3.3. Prevalence of HIV among Injecting Drug Users

For the past ten years, Tanzania has documented a significant increase in the number of people who inject drugs [27]. In the year 2012, through its outreach programs, the National AIDS Control Program (NACP) reported to have engaged 2530 individuals who were injecting drugs. Several studies have been done to assess the risk for HIV and determine the prevalence in this population in Tanzania. There was significantly higher HIV prevalence in this group compared to the general population. One study that was conducted in Dar es Salaam in 2006 reported the prevalence of 42% [12]. Among women injecting drugs, the prevalence was estimated to be 55% [28]. Studies on HIV prevalence among the members of the key populations in Tanzania are summarized in Table 1.

## 4. Risk Factors for HIV in the General Population

**Figure 1:**
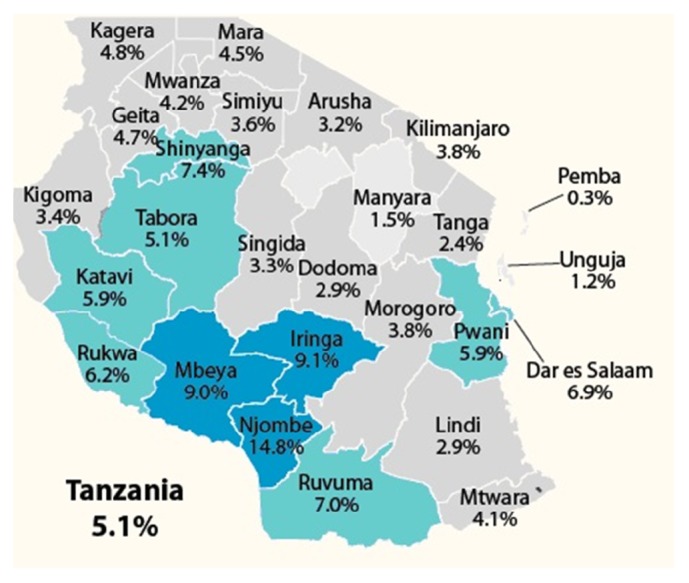
HIV prevalence among men and women aged 15–49 years in Tanzania by region [2].* Source*: Tanzania HIV/AIDS and Malaria Indicator Survey 2011/2012.

The prevalence of HIV in general population in Tanzania is estimated to be about 5.1%; however, there are geographical variations with high burden in the Southern Highland zone of the country [2] (Figure 1). Several studies have been done to determine the drivers of HIV epidemic in Tanzania. A survey that was conducted by the National Institute for Medical Research in 2009 revealed that early age for sexual debut, having multiple sexual partners, extramarital sex, and low knowledge on condoms were factors associated with HIV risk in Tanzanian communities [29]. In this survey, 85% of the respondents reported to have had their sexual debut between the ages of 10–19 years. Early sexual debut has been reported to be a predictor of having multiple sexual partners later in life [30]. Up to 25% of the respondents reported that they did not know where to get the condoms; extramarital sex was reported in 79% of the respondents. The conclusion from this survey was that high levels of multiple and concurrent sexual partners, intergenerational sex, and transactional sex were among the drivers of HIV epidemic in Tanzania.

Other studies have also demonstrated risk factors for HIV in Tanzania. A prospective study done in Mbeya found that younger age, low level of education, alcohol use, and number of sexual partners were associated with the risk for HIV [31]. The number of sexual partners was also found to be a risk factor in a case-control study done in the northwestern part of the country [32]. Another survey done in rural Tanzania found HIV prevalence of 5.6%; multiple sexual partners and alcohol consumption were associated with the risk for HIV [33].

Risky behaviors, however, are not the only reason for HIV transmission in the general population. Several studies have shown a discrepancy between high risky behavior and HIV prevalence, suggesting that there are other factors that explain HIV transmission. A study done in Zimbabwe and Tanzania found that the participants in Tanzania had more risky sexual behaviors and other risk factors for HIV than those in Zimbabwe; the prevalence of HIV was, however, higher in Zimbabweans [34]. The discrepancy between the HIV prevalence and risky sexual behavior has also been reported in another study done in four different African cities [35]. Concurrent sexual partnerships have also been believed to be among the major factors driving HIV epidemic in sub-Saharan Africa; however, one systematic review of the existing evidence concluded that concurrent sexual relationships do not explain the HIV epidemics in Africa [36].

## 5. Risky Behaviors among the Key Populations

### 5.1. Men Who Have Sex with Men

Evidence shows that same-sex sexual practices in Tanzania are not and have not been uncommon; they date as far back as the 19th century [40]. Studies done to assess sexual behaviors among MSM in Tanzania have identified several risks. A study done in Dar es Salaam, Tanzania, by Nyoni and Ross in 2013 found that condom usage was very low among the MSM. Only 43% of the participants reported using condom regularly with casual partners and 49% with regular partners in this study [16]. Inconsistent condom use was also found to be common in another survey done in Zanzibar in 2007; in this study, 85% of the respondents reported inconsistent condom use [23]. Majority of the MSM in the country have been reported to prefer receptive anal position (bottom position) [14–16] which is associated with higher risk of HIV transmission as compared to insertive anal position (top position) [41]. Paid sex and multiple sexual partners are also common in this population [14–16, 23, 37]. Another study in Dar es Salaam found out that up to 36% of respondents were practicing paid sex. The study also found that money-motivated MSM were more likely to identify themselves as bisexuals but also have significant number of partners of both sexes [37]. Having a female partner was a common finding in most of the studies [15, 23, 24] which increases the risk of transmission to the general population.

### 5.2. Female Sex Workers

The potential for female sexual workers transmitting HIV to the general population should not be underestimated [42]. Their clients can serve as the “bridging population” and transmit HIV to the general population [6]. A survey conducted in Dar es Salaam in 2010 estimated that the number of clients per female sexual worker in Tanzania averages at 3 [13]. The survey also found that female sexual workers were involved in risky sexual behaviors as reported elsewhere [6, 26] which include multiple sexual partners, inconsistent condom use, and the use of illicit drugs. With the reported high number of sexual partners and high frequency of partner change in addition to condom use being low and inconsistent, this population has very high chance of acquiring and transmitting HIV infection to their male clients who then serve as the “bridging population” to their wives and girlfriends.

### 5.3. People Who Inject Drugs (PWID)

Reports worldwide show that PWUD/PWID are at a significant risk of acquiring HIV infection when compared to the general population [43]. Their partners represent another population that can serve as a “bridging population” for HIV transmission. In 2015, UNAIDS estimated that 9% of all new infections in Eastern and Southern Africa were the clients of sex workers and other sexual partners of key populations [43]. Very few studies have been done in Tanzania to identify risky behaviors in this population. One study done in Dar es Salaam in 2014 found that this population was involved in a number of high risk behaviors that increase in their risk of acquiring HIV; however, there was low tendency for screening for HIV and other sexually transmissible infections. In this study, 37.5% of participants reported having multiple sexual partners; however, only 17% reported condom use [17]. Another study done in Mwanza, northwestern Tanzania, in 2014 also reported low condom use in this population; additionally, the study found that more than half of the participants were sharing needles and use illicit drugs during sex [39]. Needle sharing, multiple sexual partners, and low condom use were also reported in a survey done in Zanzibar [38]. Screening for HIV and other STIs was found to be low in this population in Dar es Salaam and Zanzibar [17, 38]. The role of STIs in increasing HIV transmission has been studied previously; despite some trials showing no effect in HIV transmission, most studies have shown that STIs, especially ulcerative ones, increase the risk of HIV transmission [44, 45]. Also early treatment of HIV has been shown to minimize the risk of HIV transmission [46]. The fact that this population is at increased risk for acquiring HIV with low screening rates further increases the chance of them transmitting HIV to the general population. Studies on risky behaviors among the key populations in Tanzania are summarized in Table 2.

## 6. Possible Role of Key Populations in HIV Transmission

**Figure 2:**
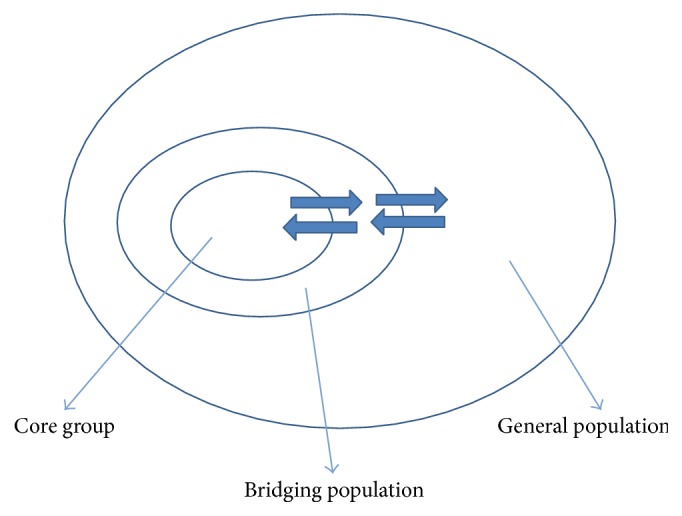
Sexual networks according to Anderson Transmission model [18].

HIV prevalence has been reported to be higher among the members of the key populations compared to the general population in Tanzania just like in other countries. As reported earlier, members of these populations have high frequency of partner change; with the high HIV prevalence, these form a “core group” in the transmission model described by Anderson [47]. According to the model, sexual partners of these individuals will serve as a “bridging population” between the members of the core groups and the general population (Figure 2). Members of the core groups and their sexual partners have been shown to be the drivers of HIV epidemic in many parts of the world [5]. Members of the key population, therefore, together with their sexual partners could be contributing to the epidemic in Tanzania.

## 7. Status of Interventions to Reduce HIV Transmission in Key Populations in Tanzania

Tanzania through scaling up HIV interventions has managed to significantly reduce the prevalence of HIV in the general population. The prevalence of HIV has been on the decline; in 2003/2004, the prevalence was estimated to be 7%. The survey done in 2007/2008 found the prevalence to be 5.7% and that in 2011/2012 found it to be 5.1%. However, the prevalence among the KPs has been found to be disproportionately high. The country therefore saw the need to have interventions among the KPs and developed a national guideline that stipulates the comprehensive package of HIV interventions for key populations [48]. The guidelines aimed at increasing access to both health-related and social services among the members of the KPs in order to minimize HIV transmission in these vulnerable and at risk groups.

The guideline among other things includes recommendations for HIV prevention, diagnosis, treatment, and care, biomedical interventions such as nonoil lubricants for MSM and medically assisted therapy (MAT) for PWID, management of comorbidities and coinfections, and finally the social and behavioral interventions for HIV prevention. The Ministry of Health committed itself to ensure implementation of this guideline by putting in place the systems and structure to support the implementation. In October 2016, the Ministry of Health decided to suspend activities for drop in centers for MSM which were responsible for differentiated service delivery for MSM; it also suspended the use of nonoil-based lubricants pending review of the existing evidence and acceptability of their use in Tanzania. The ministry aims at reviewing the package for health and HIV services for KPs and then incorporating those interventions that are internationally acceptable and in line with the country's laws, traditions, and customs.

The situation is different with PWID. Interventions for PWID have been in place before the release of the guideline. In 2010, Tanzania developed a guideline for medically assisted treatment of opioid dependence [48]; in 2011, Tanzania became the first President's Emergency Plan for AIDS Relief (PEPFAR) country in Africa to establish medically assisted therapy (MAT) as part of HIV prevention intervention for PWID by opening MAT clinic at Muhimbili National Hospital. In 2012, another pilot clinic was opened at Mwananyamala Hospital in Dar es Salaam. However, accessibility remains poor; so far such clinics are available in only one city (Dar es Salaam) in mainland Tanzania.

## 8. Conclusions and Recommendations

Members of key population have higher rates of HIV in Tanzania compared to the general population. Risky sexual behaviors have been reported to be common in these populations; interactions with the general population increase the chance of transmission of HIV to the general population. Interventions to minimize HIV prevalence and transmission risk in these populations such as promoting and providing condoms for MSM and FSW, promoting the use of water-based lubricants for MSM, screening and treating STIs, harm-reduction techniques for IDUs (e.g., needle syringe exchange programs), and HIV counseling, testing, and early treatment initiation are important measures to prevent HIV transmission in Tanzania. The country therefore needs to lay stress on the need for HIV prevention, care, and treatment among the members of the key populations.

## Figures and Tables

**Table 1:** Summary of studies on HIV prevalence among the different key populations in Tanzania.

Population	Study	Study setting	Prevalence
MSM	Ross et al., 2014 [14]	Dar es Salaam	30.2%
		Tanga	11.0%
	Dahoma et al., 2011 [23]	Zanzibar	12.3%
	Mmbaga et al., 2017 [24]	Dodoma	17.0%

FSW	NACP, 2011 [13]	Dar es Salaam	31.4%
	Kapiga et al., 2002 [25]	Moshi	26.3%

PWID	Williams et al., 2009 [12]	Dar es Salaam	42.0%
	Atkinson et al., 2011 [28]	Dar es Salaam	55.0% among women

**Table 2:** Summary on risky behaviors and the respective studies among the key populations in Tanzania.

Population	Risky behaviors	Study
MSM	Low condom use	Nyoni and Ross, 2013 [16] Dahoma et al., 2011 [23]
	Preference of receptive anal position (bottom position)	Ross et al., 2014 [14] Mmbaga et al., 2012 [15] Nyoni and Ross, 2013 [16]
	Paid sex and multiple sexual partners	Nyoni and Ross, 2013 [16] Dahoma et al., 2011 [23] Ross et al., 2014 [14] Bui et al., 2014 [37]
	Multiple sexual partners	Bui et al., 2014 [37]

FSW	Multiple sexual partners/high frequency of partner change	NACP, 2011 [13] Kapiga et al., 2002 [25]
	Inconsistent condom use	
	Alcohol and drug abuse	

PWUD/PWID	Multiple sexual partners	Mlunde et al., 2016 [17] Matiko et al., 2015 [38]
	Low and inconsistent condom use	Mlunde et al., 2016 [17] Tan et al., 2015 [39] Matiko et al., 2015 [38]
	Needle sharing	Tan et al., 2015 [39] Matiko et al., 2015 [38]
